# Reply to: The stress-inducible ER chaperone GRP78/BiP is upregulated during SARS-CoV-2 infection and acts as a pro-viral protein

**DOI:** 10.1038/s41467-022-34066-2

**Published:** 2022-11-14

**Authors:** Mohammed Samer Shaban, Christin Müller, Christin Mayr-Buro, Hendrik Weiser, M. Lienhard Schmitz, John Ziebuhr, Michael Kracht

**Affiliations:** 1 Rudolf Buchheim Institute of Pharmacology, Justus Liebig University, Giessen, Germany; 2 Institute of Medical Virology, Justus Liebig University, Giessen, Germany; 3 Institute of Biochemistry, Justus Liebig University, Giessen, Germany; 4 German Center for Lung Research (DZL), Universities of Giessen and Marburg Lung Center (UGMLC) and Cardio-Pulmonary Institute (CPI), Giessen, Germany; 5 German Center for Infection Research (DZIF), partner site Giessen-Marburg-Langen, Giessen, Germany

**Keywords:** SARS-CoV-2, Virus-host interactions, Molecular medicine

**replying to** W.-J Shin et al. *Nature Communications* 10.1038/s41467-022-34065-3 (2022)

Shaban et al.^1^ recently reported a potent antiviral effect of the ER stress inducer thapsigargin against coronaviruses that was accompanied by an increase in the major ER chaperone BiP (also called GRP78 or HSPA5), supporting the idea that elevated BiP levels may contribute to some of the drug’s antiviral effects. Beyond this, an investigation of the precise role of BiP in CoV replication was not the aim of that study. Shin et al. observed an upregulation of BiP in SARS-CoV-2-infected cells while raising concerns about some of our results. In our response, we present an additional set of quantifications that confirm our previous conclusions. In our view, a critical evaluation of the currently available evidence regarding specific BiP functions in SARS-CoV-2 infection does not allow any conclusions regarding potentially beneficial therapeutic effects in COVID-19 by pharmacological manipulation of BiP. Further experimental and pre-clinical work would be required to substantiate such a concept.

In our recent publication by Shaban et al.^1^, we observed a strong antiviral effect of the ER stress inducer thapsigargin, along with a significant and highly reproducible increase of the major ER chaperone BiP (also called GRP78 or HSPA5). This led us to propose, in the Discussion section, that strongly increased BiP levels may be involved in the antiviral effects of thapsigargin that ultimately lead to suppression of coronavirus replication. Beyond this, we did not investigate the role of BiP in CoV replication any further. We however noticed that, in most of our experiments, coronavirus infection alone was associated with a reduction in BiP protein levels (similar to the suppression of multiple other proteins of the ER stress response/unfolded protein response (UPR) as revealed by the comparison of mRNA versus protein levels of more than 100 components of the KEGG pathway hsa04141 “protein processing in endoplasmic reticulum” by RNAseq and LC-MS/MS, see Fig. 1 of Shaban et al.^1^).

**Fig. 1:**
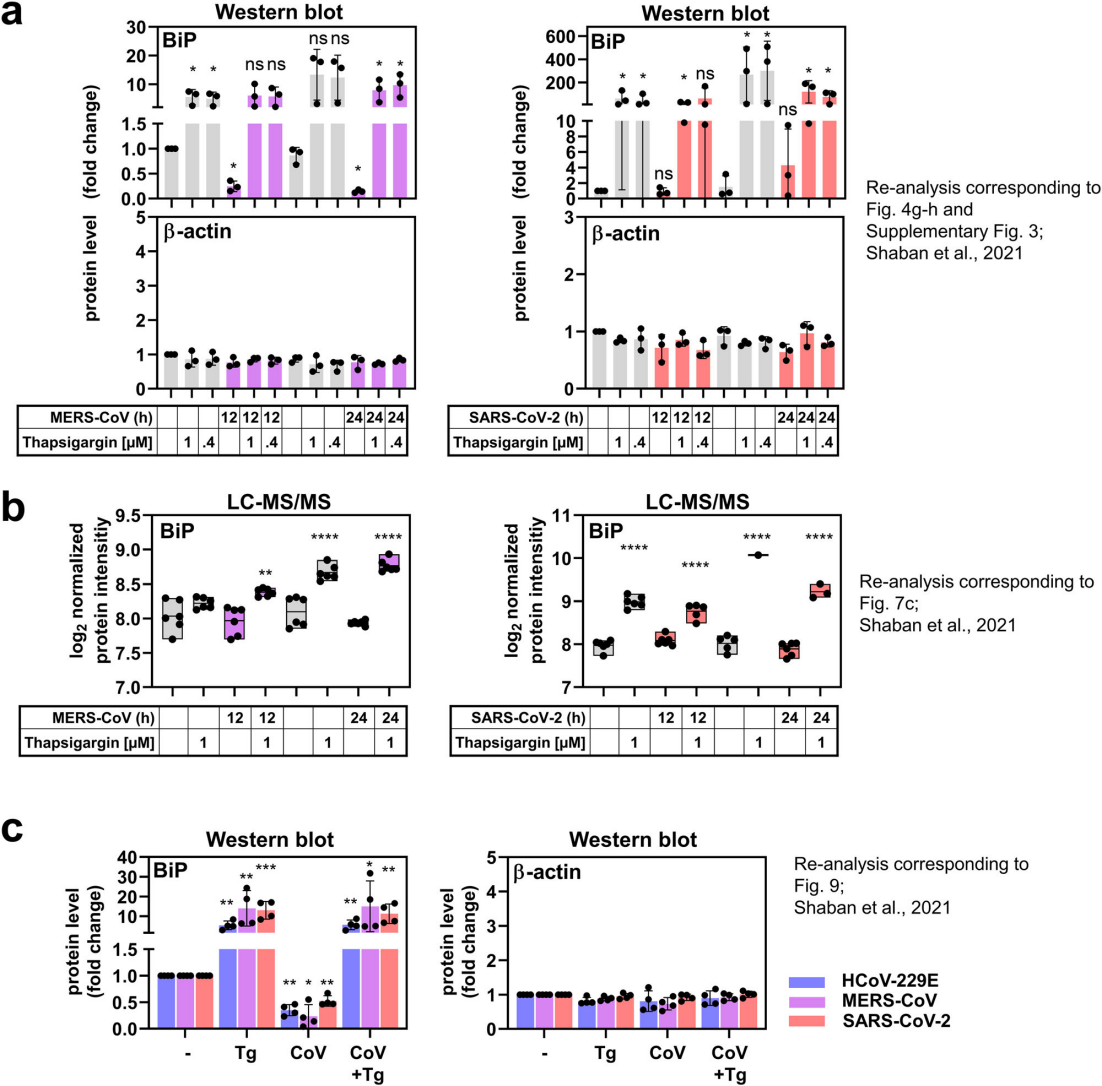
Additional quantifications of changes in BiP levels from MERS-CoV or SARS-CoV-2-infected cells in the presence or absence of thapsigargin (Tg) reported by Shaban et al.^1^. **a** Graphs with split Y-axes showing the quantification of BiP and β-actin protein bands in HuH7 cells infected with MERS-CoV (MOI of 0.5) or Vero E6 cells infected with SARS-CoV-2 (MOI of 0.5) for 12 h or 24 h as previously presented in Supplementary Fig. 3 of Shaban et al.,^1^. Data points represent three biologically independent experiments. Representative immunoblots are provided in Fig. 4 g (MERS-CoV) and Fig. 4 h (SARS-CoV-2) of Shaban et al.,^1^. BiP levels were quantified relative to the untreated control. Bar graphs show means ± s.d.; asterisks indicate significant changes (*p ≤ 0.05) obtained by two-tailed ratio-paired *t*-tests; ns indicates non-significant changes. **b** Normalized protein intensity values of BiP expression were determined by label-free LC-MS/MS-based quantification of tryptic peptides derived from total cell extracts of uninfected HuH7 cells (−), cells infected with MERS-CoV (MOI of 3) for 12 h or 24 h (left graphs), or Vero E6 cells infected with SARS-CoV-2 (MOI of 3) for 12 h or 24 h (right graphs), in the presence or absence of thapsigargin (1 µM). At least 53 (Huh7 cells) or 78 (Vero E6 cells) unique peptides were obtained, representating a sequence coverage of BiP ranging from 60.1 to 69.6%. Raw data can be retrieved using the following link: 10.6019/PXD021222. Floating bars show minimum to maximum values and means. Data points represent two biological and three technical replicates. Asterisks indicate *p* values (*p ≤ 0.05, **p ≤ 0.01, ***p ≤ 0.001, ****p ≤ 0.0001) obtained by ordinary one-way ANOVA. **c** Graphs with split *Y*-axes showing the quantification of BiP and β-actin protein bands in HuH7 cells infected with HCoV-229E (MOI of 3), MERS-CoV (MOI of 3) or Vero E6 cells infected with SARS-CoV-2 (MOI of 3) for 24 h as previously presented in Fig. 9b of Shaban et al.,^1^. Data points represent four biologically independent experiments. Representative immunoblots are shown in Fig. 9a of Shaban et al.,^1^. BiP levels were quantified relative to the untreated control. Bar graphs show means ± s.d.; asterisks indicate significant changes (*p ≤ 0.05, **p ≤ 0.01, ***p ≤ 0.001) obtained by two-tailed ratio-paired *t*-tests; ns indicates non-significant changes.

In their manuscript, Shin et al. raise concerns regarding the presentation and interpretation of BiP levels in coronavirus-infected cells, with special reference to the immunoblot data shown in Fig. 2e/f, Fig. 4b/c, Fig. 4 g/h, Fig. 9a/b, and Supplementary Fig. 3a/3b of our manuscript. To address this comment, it is important to note that we have chosen to present this particular set of immunoblot data (from a much larger set of data generated in this study) to demonstrate the very strong cell type- and virus-independent increase of BiP protein levels induced by thapsigargin in virus-infected cells, one of the key observations made in our study. To provide convincing evidence to support this major conclusion, we decided to present short exposures of the immunoblots.

Because of thapsigargin’s very strong effects on BiP levels, the BiP-specific bands in samples from nontreated/noninfected control cells and infected/nontreated cells were relatively faint. This is particularly the case for samples obtained from MERS-CoV- and SARS-CoV-2-infected cells (Fig. 4 g/h, Fig. 9, Supplementary Fig. 3). The downregulation of BiP by HCoV-229E, a less pathogenic human coronavirus, in HuH7 cells was readily detectable (Figs. 2e/f, 3e and 9a), while HCoV-229E apparently caused no change of BiP levels in human MRC-5 fibroblasts (Fig. 4b).

To support our earlier conclusions concerning BiP levels in MERS-CoV- and SARS-CoV-2-infected cells, we show again the relevant protein measurements of our study, but now focus on changes caused by coronavirus infections compared to uninfected controls (Fig. 1). We also include the normalized protein intensity values for BiP as obtained by quantitative LC-MS/MS. These additional quantification data confirm our previous conclusion that CoV infection alone does not cause an increase of BiP levels under the conditions used in our study.

In contrast, in the majority (but not all) of our experiments, CoV infection was found to cause a reduction of cellular BiP levels. The molecular changes in virus-infected cells were analyzed at 24 h p.i., taking into account that viral RNA synthesis and production of infectious virus progeny reach their maxima between 16 and 24 h p.i. while, at later time points p.i., virus titers start to decline with increasing cellular damage caused by the infection (see, for example, Fig. 3d in  Shaban et al.^1^). To obtain biologically relevant information on cellular factors and mechanisms that are critically involved in viral replication, including virus-induced changes in signaling and gene expression, it is important to study cellular changes at earlier time points p.i. and, if possible, under synchronized infection conditions (high MOI), especially if population-based assays are used. We therefore decided to infect the cells with MOIs ranging from 0.5 to 3 and analyzed the proteins at 12–24 h p.i. throughout our studies of ER stress/UPR components.

Shin et al. provide a set of immunoblot data using African green monkey kidney epithelial cells that ectopically express the SARS-CoV-2 cellular receptor, human ACE2, and human lung adenocarcinoma H1299 cells suggesting that SARS-CoV-2 infection causes increased BiP levels with a peak at late time points, i.e., 36 h p.i. (Shin et al., Fig.1a/b/e and Supplementary Fig. 1a). Also, a weak increase in GRP78 mRNA levels was observed (Shin et al., Fig. 1e, Supplementary Fig. 1b). Based on these data, the authors suggest a pro-viral role for BiP.

To further support their hypothesis, they targeted BiP levels or functions by RNAi-based and pharmacological approaches. Partial suppression of BiP by transiently transfected siRNAs resulted in a less than twofold reduction of SARS-CoV-2 spike protein levels and a reduction in viral titers of less than a log_10_ level (Shin et al., Fig. 2a–c, Supplementary Fig. 1c–e). We consider these observations as evidence for rather limited antiviral effects.

Furthermore, they treated Vero E6- ACE2 cells with a thiazole benzenesulfonamide, HA15, that was previously reported to (i) physically associate with BiP and (ii) modestly inhibit the basal ATPase activity of the chaperone at micromolar concentrations^2,3^.

The use of 2.5 µM HA15 resulted in a reduction of SARS-CoV-2 plaque numbers by ~40% (Shin et al., Fig. 2d). Surprisingly, the medium used for this experiment lacked fetal bovine serum (FBS) (see Methods by Shin et al.), probably leading to suboptimal cell growth and viral replication. There is no information as to whether the omission of FBS for several days affected SARS-CoV-2 replication in these cells and, more generally, the information provided on viral infection experiments in specific cells types does not allow an independent assessment of the data. Thus, for example, trypsin was included in the medium used to prepare SARS-CoV-2 stocks. Did this also apply to other infection experiments and, if so, did trypsin affect virus titers obtained from other cell types? More importantly, there is no data to support the potential antiviral effect of HA15 under standard cell culture conditions using medium supplemented with FBS and appropriate methods for measuring virus titers in the supernatants of cells treated with HA15 for different periods of time.

Also, there is no information on whether or not the data shown in Fig. 2d–e can be reproduced in different cell types. A major concern is the complete absence of plaques at 5 µM HA15, a just twofold higher concentration (Shin et al., Fig. 2d). In this context, it is important to note that HA15 was reported by Cerezo et al.^3^ to be a highly cytotoxic anticancer compound whose 50% inhibitory concentration (IC_50_) in A375 melanoma cells ranged between 1 and 2.5 µM, while normal human melanocytes or human fibroblasts showed little cytotoxicity at doses of up to 100 µM^3^. While results of the cell viability assays presented in Fig. 2e and Supplementary Fig. 1f suggest that Vero E6-ACE2 cells do not show cytotoxic effects at up to 5 µM HA15 when exposed to the compound alone, cell viability under infection plus HA15 treatment conditions was not assessed. Similarly, the approximately ten-fold reduction of SARS-CoV-2 RNA copies found in the lungs of hACE2-transgenic mice exposed to virus plus compound for three days can only be regarded as a preliminary, though interesting, observation, because the experiments lack the same types of controls. In our view, extensive evaluation of this model with respect to dose-response curves, time-course of infection, lung histology/pathology and mortality rates is needed. Thus, the lack of systematic side-by-side comparisons of the cytotoxicity caused by viral infection alone compared to the combined effects of SARS-CoV-2 infection and HA15 treatment constitutes a major limitation of both the in vitro and in vivo studies presented by Shin et al.

Moreover, in the study of Cerezo et al.^3^, HA15 was shown to induce several ER stress/UPR target genes and activate autophagy^2,3^. It is therefore possible that the antiviral effect proposed by Shin et al. based on the data shown in Fig. 2d-f resulted from cytotoxic or indirect effects of HA15 involving ER stress/UPR, autophagy and/or apoptosis.

In the light of these limitations, we feel that more data would be required to prove that the various biological effects of HA15 are primarily caused by specific inhibition of BiP, especially since the antiviral effects seem to be more profound compared to the transient knockdown of BiP. This would include identifying the precise mechanism of action of HA15^4^. While this compound shares some features with thapsigargin, such as the modulation of ER stress/UPR and autophagy, thapsigargin appears to be a much more potent inhibitor of coronavirus (including SARS-CoV-2) replication, as revealed by our own and two recent studies from another laboratory^1,5,6^. In these reports, specific care was taken to demonstrate that there was no additional cytotoxicity associated with thapsigargin treatment of CoV-infected cells. Moreover, antiviral effects were observed well below the cytotoxic effects caused by thapsigargin, resulting in high selectivity indices (reviewed in ref. 7), while similarly favorable antiviral efficacy and selectivity profiles remain to be demonstrated for HA15.

Shin et al. cite four publications in support of a (pro-viral?) role of BiP in SARS-CoV-2 infection^8–11^. These studies were performed across widely diverging experimental and infection conditions. For example, in the study of Echavarria-Consuegra et al.^9^, the mouse hepatitis virus (MHV, a betacoronavirus) was reported to downregulate BiP protein levels in murine fibroblast 17 Cl-1 cells (Fig. 2f in ref. 9), while SARS-CoV-2 weakly induced BiP in Vero CCL81 or human lung adenocarcinoma Calu3 cells (Fig. 5a/b in ref. 9). In the study by Sims et al., BiP was induced by MERS-CoV in human lung microvascular endothelial cells but not in fibroblasts^10^. Importantly, a functional role of BiP for CoV replication in these systems was not demonstrated^9,10^. Taken together, these studies lead us to conclude that the information available to date does not suffice to establish a coherent model of BiP regulation and function in CoV infection.

Of note, several recent genome-wide sgRNA screens failed to identify BiP as a relevant factor in SARS-CoV-2 replication (reviewed in ref. 12). In the absence of further strong mechanistic (or at least functional) evidence from carefully controlled loss- or gain-of-function experiments proving the specific role(s) of BiP for SARS-CoV-2 replication, it seems premature to suggest a specific role for BiP as a pro-viral (or anti-viral) factor.

As an abundant ER chaperone and ER stress sensor, BiP plays multiple roles in stress, infection and immunity (reviewed in refs. 13, 14, 15, 16, 17). Membrane-associated BiP supports entry of several viruses into cells and BiP levels are increased in the blood of COVID-19 patients^18–20^. Thus, BiP may serve as a useful biomarker to judge the severity of (viral) infections. However, given its broad spectrum of activities under various conditions it is difficult to link BiP inhibition with a specific benefit that may be used in strategies to reduce the risk of SARS-CoV-2 infection or severe outcomes of COVID-19. Specifically, suppression of BiP or BiP activity may turn out to be a double-edged sword in RNA virus infection: even if BiP inhibition was shown to have some antiviral effect, this effect would probably come at a cost because the toxic stress caused by accumulating misfolded proteins^21–23^ may outweigh the beneficial antiviral effects of BiP inhibitors.

In conclusion, regardless of whether BiP levels are differentially regulated in different infection models or at different time points p.i., there is, in our opinion, currently not enough evidence to suggest that a combined treatment using established anti SARS-CoV-2 therapeutics (or emerging compounds such as thapsigargin) and anti-GRP78 (BiP) compounds will have an additive or synergistic effect in suppressing SARS-CoV-2 replication. We consider it more likely that this sort of combination therapy results in increased cytotoxicity. Clearly, more experimental and pre-clinical work is needed to support this concept.

## Methods

### Cells and viruses

Viruses and cell sources between the two studies differ. Specifically, in the study by Shaban et al.^1^, the following cell lines and virus strains were used: HuH7 human hepatoma cells (Japanese Collection of Research Bioresources cell bank), which were maintained in Dulbecco’s modified Eagle’s medium (DMEM) complemented with 10% filtrated bovine serum (FBS Good Forte; PAN Biotech, Cat No. P40-47500), 2 mM L-glutamine, 100 U/ml penicillin and 100 μg/ml streptomycin and Vero E6 African green monkey kidney epithelial cells (ATCC CRL-1586), which were grown in DMEM, 10% FBS, 100 U/ml penicillin, and 100 μg/ml streptomycin.

Genome sequences of coronavirus strains were as follows: HCoV-229E (NCBI accession number AF304460.1, NCBI reference sequence NC_002645.1), MERS-CoV (NCBI accession number JX869059, NCBI reference sequence NC_01984 3.3). SARS-CoV-2 (NCBI Short Read Archive repository under bioproject PRJNA658242 (SRA accession number SRX9907172 and SRX8975039). MERS-CoV and SARS-CoV-2 were kindly provided by Christian Drosten.

All further details are available in Shaban et al.^1^.

### Protein analyses

Cell lysis, protein extraction, Western blotting and mass spectrometry-based proteomics analyses are described in detail in Shaban et al.^1^.

### Statistics, quantification, and reproducibility

Quantification of data and statistical parameters were calculated using GraphPad Prism 5.0, 8.4.3, or 9.4.1, Perseus (versions 1.6.10.50 (MERS-CoV) or 1.6.14 (SARS-CoV-2)), ImageLab (versions 5.2.1 or 6.0.1) and Microsoft Excel 2016. Further details are given in Shaban et al.^1^.

### Reporting summary

Further information on research design is available in the Nature Research Reporting Summary linked to this article.

## Supplementary information

Reporting Summary

## Data Availability

Source data are provided with this paper. Additional source data (immunoblot images, data sets of LC-MS/MS experiments) have been previously published^1^ and are available under the following link: https://www.nature.com/articles/s41467-021-25551-1#Sec18. Mass spectrometry raw data can be retrieved using the following link: 10.6019/PXD021222. Source data are provided with this paper.

## Source data

Source Data
