# Supplementary material for: Reply to: The stress-inducible ER chaperone GRP78/BiP is upregulated during SARS-CoV-2 infection and acts as a pro-viral protein
Source: Nat Commun. 2022 Nov 14;13:6550. doi: 10.1038/s41467-022-34066-2 (PMC9663517; doi:10.1038/s41467-022-34066-2)
Supplement: Supplementary file 1 — Reporting Summary [file 41467_2022_34066_MOESM1_ESM.pdf]

nature research

NCOMMS-22-00163B

Corresponding author(s): Michael Kracht

Last updated by author(s): 05_10_2022

Reporting Summary

Nature Research wishes to improve the reproducibility of the work that we publish. This form provides structure for consistency and transparency in reporting. For further information on Nature Research policies, see our Editorial Policies and the Editorial Policy Checklist.

Statistics

For all statistical analyses, confirm that the following items are present in the figure legend, table legend, main text, or Methods section.

n/a	Confirmed	
☐	☒	The exact sample size (*n*) for each experimental group/condition, given as a discrete number and unit of measurement
☐	☒	A statement on whether measurements were taken from distinct samples or whether the same sample was measured repeatedly
☐	☒	The statistical test(s) used AND whether they are one- or two-sided<br>*Only common tests should be described solely by name; describe more complex techniques in the Methods section.*
☒	☐	A description of all covariates tested
☒	☐	A description of any assumptions or corrections, such as tests of normality and adjustment for multiple comparisons
☐	☒	A full description of the statistical parameters including central tendency (e.g. means) or other basic estimates (e.g. regression coefficient) AND variation (e.g. standard deviation) or associated estimates of uncertainty (e.g. confidence intervals)
☐	☒	For null hypothesis testing, the test statistic (e.g. *F*, *t*, *r*) with confidence intervals, effect sizes, degrees of freedom and *P* value noted<br>*Give P values as exact values whenever suitable.*
☒	☐	For Bayesian analysis, information on the choice of priors and Markov chain Monte Carlo settings
☒	☐	For hierarchical and complex designs, identification of the appropriate level for tests and full reporting of outcomes
☒	☐	Estimates of effect sizes (e.g. Cohen's *d*, Pearson's *r*), indicating how they were calculated

Our web collection on statistics for biologists contains articles on many of the points above.

Software and code

Policy information about availability of computer code

Data collection	*Provide a description of all commercial, open source and custom code used to collect the data in this study, specifying the version used OR state that no software was used.*
Data analysis	Quantification of data and statistical parameters were calculated using GraphPad Prism 5.0, 8.4.3, or 9.4.1, Perseus (versions 1.6.10.50 (MERS-CoV) or 1.6.14 (SARS-CoV-2)), ImageLab (versions 5.2.1 or 6.0.1) and Microsoft Excel 2016. Further details are given in Shaban et al. 1.

For manuscripts utilizing custom algorithms or software that are central to the research but not yet described in published literature, software must be made available to editors and reviewers. We strongly encourage code deposition in a community repository (e.g. GitHub). See the Nature Research guidelines for submitting code & software for further information.

Data

Policy information about availability of data

All manuscripts must include a data availability statement. This statement should provide the following information, where applicable:

- Accession codes, unique identifiers, or web links for publicly available datasets
- A list of figures that have associated raw data
- A description of any restrictions on data availability

The mass spectrometry proteomics data in this study shown in Fig. 1b have been deposited to the ProteomeXchange Consortium (Deutsch et al, 2020) via the PRIDE partner repository (Perez-Riverol et al, 2019) with the dataset identifier PXD021222 (http://dx.doi.org/10.6019/PXD021222). The remaining data generated in this study are provided in the Supplementary Information / Source Data files. Source data are provided with this paper.

Field-specific reporting

Please select the one below that is the best fit for your research. If you are not sure, read the appropriate sections before making your selection.

☒ Life sciences ☐ Behavioural & social sciences ☐ Ecological, evolutionary & environmental sciences

For a reference copy of the document with all sections, see nature.com/documents/nr-reporting-summary-flat.pdf

Life sciences study design

All studies must disclose on these points even when the disclosure is negative.

Sample size	No sample size calculation was performed using statistical methods.
Data exclusions	No data were excluded.
Replication	The number of biologically independent replicates and individual data points are indicated in the figure legends.
Randomization	No randomization was performed, as this was not applicable to our study.
Blinding	Blinding was not relevant to the study.

Reporting for specific materials, systems and methods

We require information from authors about some types of materials, experimental systems and methods used in many studies. Here, indicate whether each material, system or method listed is relevant to your study. If you are not sure if a list item applies to your research, read the appropriate section before selecting a response.

Materials & experimental systems

n/a	Involved in the study	
☐	☒	Antibodies
☐	☒	Eukaryotic cell lines
☒	☐	Palaeontology and archaeology
☒	☐	Animals and other organisms
☒	☐	Human research participants
☒	☐	Clinical data
☒	☐	Dual use research of concern

Methods

n/a	Involved in the study	
☒	☐	ChIP-seq
☒	☐	Flow cytometry
☒	☐	MRI-based neuroimaging

Antibodies

Antibodies used

anti β-actin (Santa Cruz, #sc-47778, 1:1000), anti BiP (Cell Signaling, #3177, 1:1000).
The following secondary antibodies were used: polyclonal goat anti mouse immunoglobulins/HRP (Dako P0447, 1:2000), polyclonal goat anti rabbit immunoglobulins/HRP (Dako, P0448, 1:2000).

Validation

1) Anti-β-actin (C4) mouse Monoclonal IgG1 Santa Cruz Sc-47778 C0916 et al. WB; IP; IHC (P); IF; ELISA pri. WB: 1:1000
sec. WB: 1:10000
2) Anti-BiP (C50B12) rabbit Monoclonal IgG Cell Signaling 3177 10 FC; IHC (P); WB WB: 1:1000

Primary antibodies for WB were diluted in TBS-T w/ 5% dry milk, if not indicated otherwise.
Secondary antibodies for WB were diluted 1:2000 in TBS-T w/ 5% dry milk, if not indicated otherwise.

Legend: WB/IB = WesternBlot/Immunoblotting; IP = Immunoprecipitation; ICH (P)/(F) = Immunohistochemistry (Paraffin/frozen); ICC/IF = Immunocytochemistry/Immunofluorescence

Eukaryotic cell lines

Policy information about cell lines

Cell line source(s)

HuH7 human hepatoma cells: Japanese Collection of Research Bioresources (JCRB) cell bank (Nakabayashi et al, 1982)
Vero E6 African green monkey kidney epithelial cells (ATCC CRL-1586)

Authentication

No authentification was performed.

Mycoplasma contamination	HuH7 cells were confirmed to be free of mycoplasma using the Venor® GeM Classic kit (Minerva Biolabs).
Commonly misidentified lines<br>(See ICLAC register)	According to our knowledge the cell lines used in our study do not belong to misidentified cell lines; they are not contained in the currently available version of the ICLAC register.
